# Unravelling the Biological Activities of the *Byttneria pilosa* Leaves Using Experimental and Computational Approaches

**DOI:** 10.3390/molecules25204737

**Published:** 2020-10-15

**Authors:** Mifta Ahmed Jyoti, Niloy Barua, Mohammad Shafaet Hossain, Muminul Hoque, Tahmina Akter Bristy, Shabnur Mahmud, Kamruzzaman, Md. Adnan, Md. Nazim Uddin Chy, Arkajyoti Paul, Mir Ezharul Hossain, Talha Bin Emran, Jesus Simal-Gandara

**Affiliations:** 1 Department of Pharmacy, International Islamic University Chittagong, Chittagong 4318, Bangladesh; mifta_ahmed@yahoo.com (M.A.J.); niloybaruaniloy@gmail.com (N.B.); bappyshafaet@gmail.com (M.S.H.); muminul359@gmail.com (M.H.); tahminabristy4@gmail.com (T.A.B.); mahmudshilpi56@gmail.com (S.M.); kamruzzamanarafat@gmail.com (K.); mdadnan1991.pharma@gmail.com (M.A.); nazim107282@gmail.com (M.N.U.C.); 2 Department of Theoretical and Computational Chemistry, University of Dhaka, Dhaka 1000, Bangladesh; 3 Department of Bio-Health Technology, Kangwon National University, Chuncheon 24341, Korea; 4 Department of Pharmacy, BGC Trust University Bangladesh, Chittagong 4381, Bangladesh; arka.bgctub@gmail.com; 5 Nutrition and Bromatology Group, Department of Analytical and Food Chemistry, Faculty of Food Science and Technology, University of Vigo—Ourense Campus, E32004 Ourense, Spain

**Keywords:** *Byttneria pilosa*, anti-inflammatory, analgesic, anxiolytic, neuropharmacological, anti-diarrheal

## Abstract

*Byttneria pilosa* is locally known as Harijora, and used by the native hill-tract people of Bangladesh for the treatment of rheumatalgia, snake bite, syphilis, fractured bones, elephantiasis and an antidote for poisoning. The present study was carried out to determine the possible anti-inflammatory, analgesic, neuropharmacological and anti-diarrhoeal activity of the methanol extract of *B. pilosa* leaves (MEBPL) through in vitro, in vivo and in silico approaches. In the anti-inflammatory study, evaluated by membrane stabilizing and protein denaturation methods, MEBPL showed a significant and dose dependent inhibition. The analgesic effect of MEBPL tested by inducing acetic acid and formalin revealed significant inhibition of pain in both tests. During the anxiolytic evaluation, the extract exhibited a significant and dose-dependent reduction of anxiety-like behaviour in mice. Similarly, mice treated with MEBPL demonstrated dose-dependent reduction in locomotion effect in the open field test and increased sedative effect in the thiopental sodium induced sleeping test. MEBPL also showed good anti-diarrheal activity in both castor oil induced diarrheal and intestinal motility tests. Besides, a previously isolated compound (beta-sitosterol) exhibited good binding affinity in docking and drug-likeliness properties in ADME/T studies. Overall, *B. pilosa* is a biologically active plant and could be a potential source of drug leads, which warrants further advanced study.

## 1. Introduction

Arthritis is a fundamental autoimmune disease, causes chronic inflammation in the joints’ connective tissue, leads to progression of synovial inflammation and cartilage destruction [1]. Many researchers reported that pro-inflammatory mediators like tumour necrosis factor (TNF)-alpha, interleukin (IL)-2 and enzymes are the potential stimulators of developing persistent and multiple disorders [2]. Nevertheless, a direct or indirect relationship between arthritis and these mediators is not surprising. To lessen the rheumatoid arthritis, various non-steroidal anti-inflammatory drugs (NSAIDs) (e.g., aspirin, ibuprofen, indomethacin, etc.) are being used clinically, but have several limitations (i.e., insufficient therapeutic interventions) and undesired side effects, such as amnesia, memory disturbance, drowsiness and sexual dysfunction.

Similar to arthritis, pro-inflammatory mediators are also the common etiology of persistent pain. Different sorts of conventional therapy were implemented to relieve pain since primitive times. Recently, various kinds of pain killers have been introduced which are now emerging as intolerant for the management of pain [3]. The manifestation of the addictive properties and unwanted side effects of presently used anti-nociceptive drugs have restricted their long-term application [4]. In this regard, traditional medicinal from indigenous knowledge can be safe therapeutic agents. Therefore, exploration of novel bioactive compounds from medicinal plants can play a pivotal role in the discovery and development of a sustainable anti-nociceptive agent.

Pain and inflammation are another important dominant factor for mental and behavioural disarrays [5]. Several neurotransmitters, including norepinephrine and serotonin have parallel expression pathways for both nociception and neuropsychiatric disorders [6]. Thus, chronic pain and inflammation can contribute to developing anxiety-like complications [7]. Some sedatives and hypnotic drugs are used to treat anxiety by persuading sleep inception. However, serious side effects like respiratory, digestive, immune system dysfunctions, physical dependence and tolerance are appearing due to regular use of currently obtainable sedative-hypnotic medications [8]. Henceforth, it is extremely needed to develop a new sedative-hypnotic drug with less adverse effect to managing various psychiatric disorders.

Local inflammation in the intestine generally stimulates prostaglandin biosynthesis which produces an intestinal dysfunction by suppressing the ions and water reabsorption [9,10]. This deleterious effect leads to diarrhoea by manifesting in frequent bowel movement with wet stool and abdominal cramps. At present, diarrhoea is regarded as a life-threatening disease due to the substantial report of paediatric morbidity and mortality throughout the world. Diarrhoea severely strikes malnourished children in developing countries due to the lack of useful drug and upsurge in resistance to antibiotics [11]. Consequently, safe and effective drugs from plant origins are being searched for that might be a vital source of alternative therapies.

*Byttneria pilosa* Roxb. is locally known as Harijora, which belongs to the Sterculiaceae family. Due to its medicinal benefits, *B. pilosa* is very well-known among tribal communities (Chakma, Marma and Khumi) of Bangladesh [12]. This plant was commonly found in Chittagong (specially hill tracts and forests area), Cox’s Bazar and Sylhet, Bangladesh. The plant is a large woody climber being mortised, setaceous and having twigs. The leaves of the plant are suborbicular, palmately 3-lobed, and furry on both surfaces [13]. The crushed stems of *B. pilosa* are applied for the treatment of boils, rheumatalgia, snake bite and syphilis. The Khumi community uses the tender stem paste for the treatment of fractured bones. In the Tripura community, a paste of root is applied in affected areas for the treatment of elephantiasis. In addition, juice from the root is used as an antidote in cases of poisoning. The plant also produces foam which is irritating to the eyes [14]. A compound named beta-sitosterol was isolated from the roots of the *B. pilosa* by Fourier-transform infrared spectroscopy (FTIR) analysis and physicochemical properties.

However, despite such popularity and the medicinal value of this plant, to date there is still lack of scientific validation on the phytochemical and pharmacological points of view. Therefore, we have aimed to evaluate the presence of anti-inflammatory, analgesic, anxiolytic, locomotion, sedative and anti-diarrheal activity of the methanol extract of *B. pilosa* leaves. As one compound was identified for the plant by FTIR analysis, we carried out computational studies by using molecular docking, PASS prediction and ADME properties analysis.

## 2. Results and Discussion

Humans have been using natural-derived medicines since primitive times to prevent and treat different diseases [15]. As a natural assortment, plant-derived medicines are the crucial wellspring of possibly helpful structures for the development of new compounds which could lead to establishing safe drugs [16]. The physiological and therapeutic activities of a plant extract are disclosed through the phytochemical analysis. The presence of numerous phyto-constituents increases the possibility of having different kinds of therapeutic activities like anti-inflammatory, anti-nociceptive, antiviral, antimicrobial, anti-diarrheal, thrombolytic and so on. An important phytoconstituent “alkaloids” is very beneficial for the treatment of different disorders of both humans and animals [17]. Phenolic compounds are reported to have anti-nociceptive, antioxidant, anti-inflammatory and anti-proliferative activities [18,19]; terpenoids are found to have antimicrobial, nematocidal, potent anti-hypertensive, anti-inflammatory, insecticidal and anti-parasitic properties [20]; and flavonoids are responsible for analgesic, anti-diarrheal, antioxidant, anti-inflammatory, anti-allergic, antibacterial, antiviral and anticancer activities [21]. In our study, the analysis of qualitative phytoconstituents of MEBPL revealed that various secondary metabolites are present like alkaloids, carbohydrates, glycosides, flavonoids, phenols, tannins, saponins, terpenoids, fixed oil, quinones and resins, but the absence of cholesterol and protein (Table 1). These beneficial secondary metabolites provide diverse biological actions with state-of-the-art therapeutic interventions. However, by considering the phytochemical and ethnomedicinal value of this plant we scientifically explored the possible pharmacological potentials of *B. pilosa*.

### 2.1. Effect of MEBPL on Anti-Inflammatory Activity

**Figure 1:**
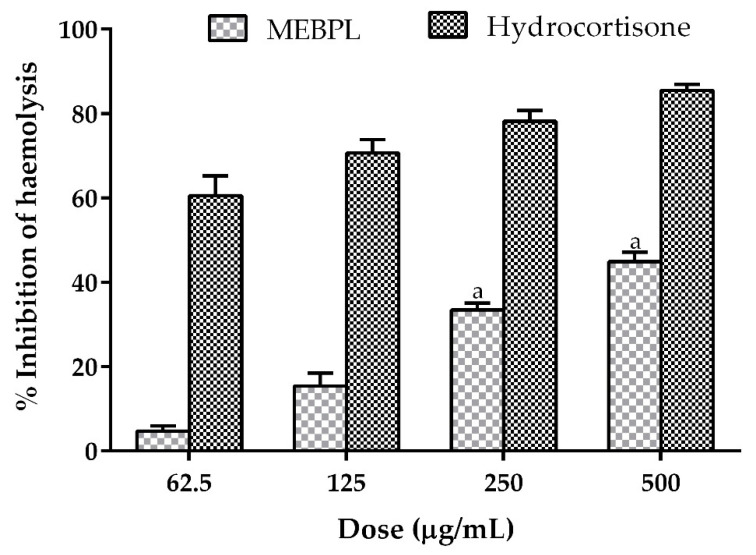
Percentages of inhibition of haemolysis of the erythrocyte membrane by methanol extract of *B. pilosa* leaves (MEBPL) and standard drug hydrocortisone. Results are mean ± SEM (*n* = 3). ^a^ *p* < 0.05, significantly different from control.

The possible anti-inflammatory effect of MEBPL was undertaken by membrane stabilization and protein denaturation assay. Lysosomal lysis and enzyme discharging are caused by the inflammation, thus, a different kind of disorder. The NSAIDs possess their remedial effect either by stabilizing lysosomal membranes or by preventing the release of enzymes from lysosomes [22]. There are similarities between human red blood cell (HRBC) membranes and lysosomal membranes, as HRBC membranes may be lysed by the use of an injurious agent (e.g., phenyl-hydrazine), a hypotonic medium and heat. In our study, dose dependent haemolysis inhibition of MEBPL was found and the protection of haemolysis persuaded by the hypotonic solution significantly (*p* < 0.05) increased compared to the control. The percentages of inhibition of haemolysis were 4.67 ± 1.24%, 15.41 ± 3.04%, 33.44 ± 1.61% and 44.88 ± 2.27% for the extract, whereas the standard hydrocortisone exhibited inhibition of 60.56 ± 2.67%, 70.66 ± 1.83%, 78.17 ± 1.47% and 85.45 ± 0.84%, respectively, at the doses of 62.5, 125, 250 and 500 μg/mL (Figure 1). This outcome demonstrated a potential HRBC haemolysis inhibitory effect of MEBPL, indicating anti-inflammatory insight for the plant extract.

**Figure 2:**
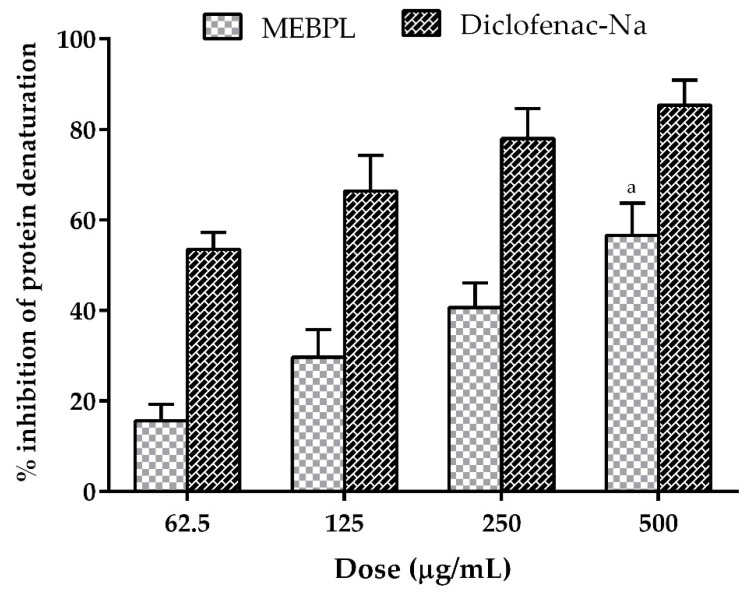
Percentages of protein denaturation by methanol extract of *B. pilosa* leaves (MEBPL) and standard drug diclofenac sodium. Values are mean ± SEM (*n* = 3). ^a^ *p* < 0.05, significantly different from control.

The chronic inflammatory response, such as arthritis, causes protein denaturation. The key mechanism of action of NSAIDs is to provide protection against denaturation of protein and play an important role in the treatment of inflammation [23]. To determine such comparable action, we conducted tests of the protein denaturation inhibitory effect of different doses of MEBPL (Figure 2). The extract exhibited a significant (*p* < 0.05) and dose dependent anti-inflammatory effect. The highest percentage of inhibitory activity was found of 56.57 ± 4.11% at the dose of 500 μg/mL for the extract; while at the same dose standard the drug diclofenac sodium produced 85.32 ± 3.22% inhibition. The ability to inhibit hypotonic protein denaturation by MEBPL may provide a significant contribution to the anti-inflammatory properties. As a result, the extract exerts anti-inflammatory activity, as it gives significant protection against HRBC membrane lysis and provides reduction of protein denaturation. Several researches reported that presence of various phytoconstituents such as phenols, terpenoids and flavonoids [24] which might be accountable for the anti-inflammatory activities of the plant.

### 2.2. Effect of MEBPL on Analgesic Activity

Nociception can cause oedema, leukocyte eruption and inflammation, which is activated or triggered by releasing pro-inflammatory mediators. The manifestation of abdominal constriction (writhing response) after inducing acetic acid (intraperitoneally) into the mice is a well-designed model for the study of visceral pain. This animal model also helps to evaluate the peripherally analgesic effect of the plant extract [25]. In this test, pain sensation is perceived by producing pro-inflammatory endogenous mediators (prostaglandins, cytokines, histamine, lipoxygenase, cyclooxygenase and serotonin) in the peripheral tissue which not only influence the stimulation of prostaglandin but also enhance the capillary permeability progresses of inflammatory ache [26]. Analgesics are the chemical substances that lower the amount of writhing by inhibiting prostaglandin synthesis [27]. In the acetic acid writhing test, the MEBPL exposed a significant and dose dependent inhibition in the nociceptive nerve. The MEBPL respectively exhibited 24.73% and 33.87% inhibition of writhing response at 200 and 400 mg/kg doses, while the reference drug diclofenac sodium (10 mg/kg) showed 65.59% inhibition compared to the control (Table 2).

Besides, the formalin induced licking test is a biphasic technique for both centrally and peripherally anti-nociceptive pain mediated by two different mechanisms. The early phase is related to the neurogenic pain and the late phase is due to the inflammatory pain. The NSAIDs inhibit the late phase whereas the narcotic drugs inhibit both phases [28]. Here, MEBPL provided a dose dependent reduction of paw licking time in early phase at doses of 200 and 400 mg/kg (Table 3). However, compared to the late phase of the control, a dose of 400 mg/kg significantly (*p* < 0.001) decreased latency to uneasiness. On the other hand, standard drugs decreased the time of paw licking significantly at both phases. Such significant inhibition in the analgesic activity exhibited by the plant might be due to the availability of several bioactive phytoconstituents such as alkaloids, phenols [18], tannins [29,30] and flavonoids.

### 2.3. Effect of MEBPL on Anxiolytic Activity

The hole-board test model used to exhibit the anxiolytic activity of the extract. After administration of the extract, mice with increased of head dipping behaviour reflect as anxiolytic whereas hesitancy reflects anxiogenic state during the hole-board test. Generally anxiolytic drugs impose their activity by binding with the gamma aminobutyric acid (GABA) receptor in the brain, increasing GABAergic neurotransmission, as the GABA receptor is important for balancing the neural inhibition as well as excitation [31]. In our experiment, the anxiolytic activity of MEBPL tested by hole-board test in mice is presented in Table 4. The dose of 400 mg/kg of the extract exhibited significant (*p* < 0.01) increase in the number of head dipping (40.20 ± 2.82) compared to the control, while the standard drug diazepam 1 mg/kg showed 58.20 ± 4.02 number of head dipping. It is observed in our study that the head dipping behaviour of mice after the treatment of the extract increases as the dose is raised from 200 to 400 mg/kg, which revealed the anxiolytic potentiality of the extract.

### 2.4. Effect of MEBPL on Locomotor Activity

**Figure 3:**
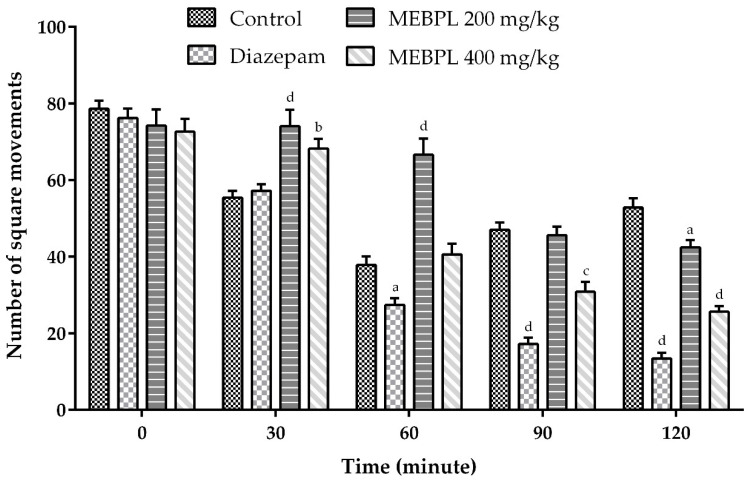
Locomotor effect of methanol extract of *B. pilosa* leaves (MEBPL) using open field test in mice. Values are stated as mean ± SEM (*n* = 5). ^a^ *p* < 0.05, ^b^ *p* < 0.01, ^c^ *p* < 0.001 and ^d^ *p* < 0.0001 compared with control group followed by Dunnett’s test of one-way ANOVA (GraphPad Prism 6.0).

During the open field test, mice treated with MEBPL manifested central nervous system (CNS) depressive activity, such as decreases in exploration and locomotion. Diazepam is a drug of the benzodiazepine group and acts as a central sensory depressant through binding with the GABA receptor, moderates excitement and calms the recipient. Quantitatively, the most excitatory and inhibitory neurotransmitters are GABA and glutamate [32]. Therefore, receptors regarding these two neurotransmitters always seem to be important targets for psychotropic drugs. Here MEBPL (200 and 400 mg/kg) significantly and dose dependently reduced the square movements in the open field test of locomotion activity (Figure 3). The number of squares travelled by the mice at dose 400 mg/kg was decreased significantly (*p* < 0.001) at all intervals over 120 min compared to the control group.

### 2.5. Effect of MEBPL on Sedative Activity

**Figure 4:**
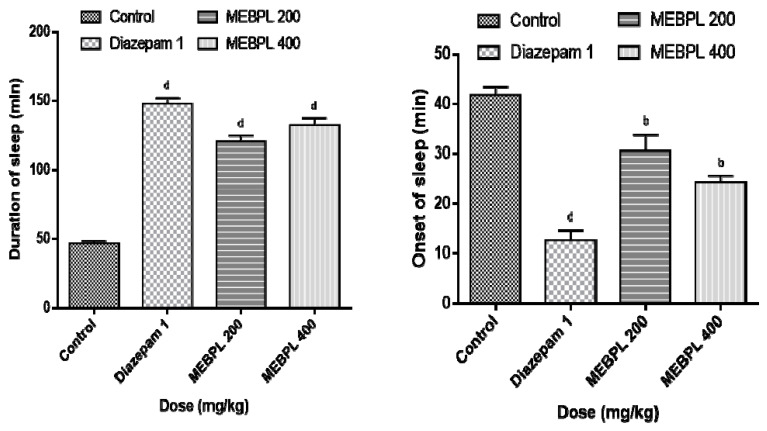
Sedative effect of methanol extract of *B. pilosa* leaves (MEBPL) by using thiopental sodium induced sleeping time test. Values are stated as mean ± SEM (*n* = 5). ^b^ *p* < 0.01 and ^d^ *p* < 0.0001 compared with control group followed by Dunnett’s test of one-way ANOVA (GraphPad Prism 6.0).

In the sedative activity test, the extract significantly (*p* < 0.01) and dose dependently showed reduction in the onset time of falling asleep and for longer duration compared to the control (Figure 4). The length of sleeping time at the doses of 200 and 400 mg/kg was 120.59 ± 4.23 min and 132.31 ± 5.05 min, respectively, whereas the reference standard drug exhibited 147.79 ± 3.97 min sleeping. Diazepam (standard drug) produces anxiolytic as well as a sedative effect, as it minimizes the onset of rest and extended the duration of rest as well as reduced exploratory action [33]. Similar response was observed after the treatment of MEBPL, which might be hyperpolarisation of the membrane and causes the CNS depressive effect, thus leading to sedative activity.

### 2.6. Effect of MEBPL on Anti-Diarrheal Activity

Castor oil, its most active metabolite (ricinoleic acid), is regarded as a well-known diarrheal agent. Irritation of the intestinal mucosa is attempted when ricinoleic acid is released from castor oil by the action of the lipase enzyme, this causes secretion of the prostaglandin and nitric oxide (inflammatory mediators) that lead to stimulation of the intestinal motility by suppressing electrolyte and water reabsorption [34]. However, to unveil the anti-diarrheal effect of MEBPL, we conducted the castor-oil induced diarrheal test on mice. As shown in Table 5, the extract expressively inhibited diarrhoea and defecation in a dose dependent way compared to the control group. In fact, a 400 mg/kg dose of the extract exposed more significant inhibition (*p* < 0.0001) in defecation and diarrhoea which was higher than the reference drug loperamide 5 mg/kg.

Besides, MEBPL showed significantly reduction in the peristalsis index. The dose of 400 mg/kg exhibited noteworthy reduction of peristalsis index by 51.30 ± 5.04% (*p* < 0.001), while the standard loperamide (5 mg/kg) revealed 45.33 ± 2.27% (*p* < 0.0001) reduction. Furthermore, a 400 mg/kg dose showed 49.13% inhibition of intestinal motility, whereas the reference loperamide (5 mg/kg) exhibited 54.51% inhibition compared to the control. The intestinal motility effect of MEBPL by inducing castor-oil summarized in Table 6. However, the observed anti-diarrheal activity by the MEBPL could be the availability of secondary metabolites like flavonoids and tannins which can cause death of the worms in the gastrointestinal tract by selectively binding with free proteins [35].

### 2.7. In Silico Studies

**Figure 5:**
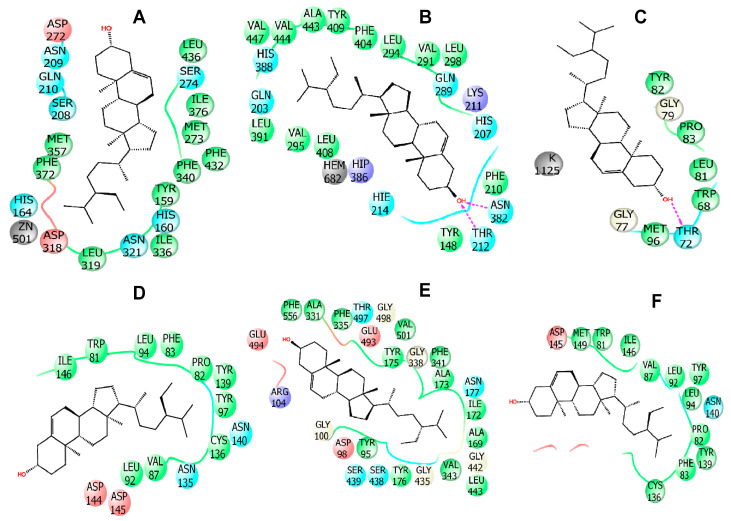
The best 2D representation found of the interactions between beta-sitosterol with (**A**) PDE4 enzyme (PDB ID: 4WCU), (**B**) COX-2 enzyme (PDB ID: 6COX), (**C**) potassium channel (PDB ID: 4UUJ), (**D**) bromodomain of human BRD4 in complex with midazolam (PDB ID: 3U5K), (**E**) serotonin transporter (PDB ID: 5I6X), and (**F**) bromodomain of human BRD4 in complex with alprazolam (PDB ID: 3U5J).

**Figure 6:**
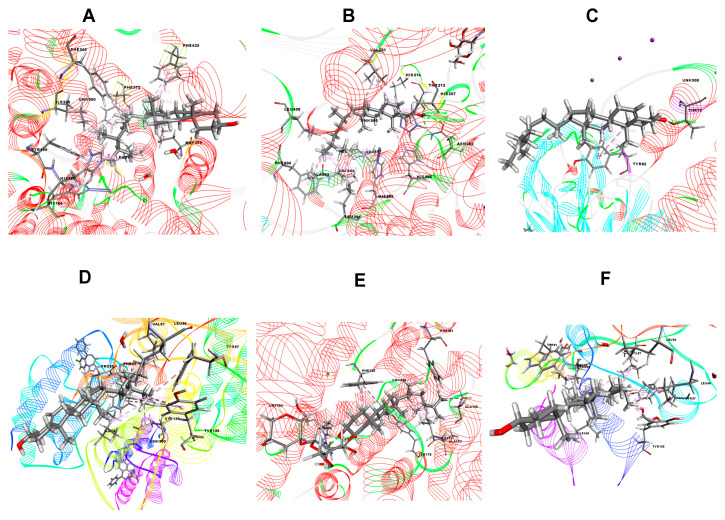
Best representations of beta-sitosterol in the binding pocket of (**A**) PDE4 enzyme (PDB ID: 4WCU), (**B**) COX 2 enzyme (PDB ID: 6COX), (**C**) potassium channel (PDB ID: 4UUJ), (**D**) bromodomain of human BRD4 in complex with midazolam (PDB ID: 3U5K), (**E**) serotonin transporter (PDB ID: 5I6X), and (**F**) bromodomain of human BRD4 in complex with alprazolam (PDB ID: 3U5J).

**Figure 7:**
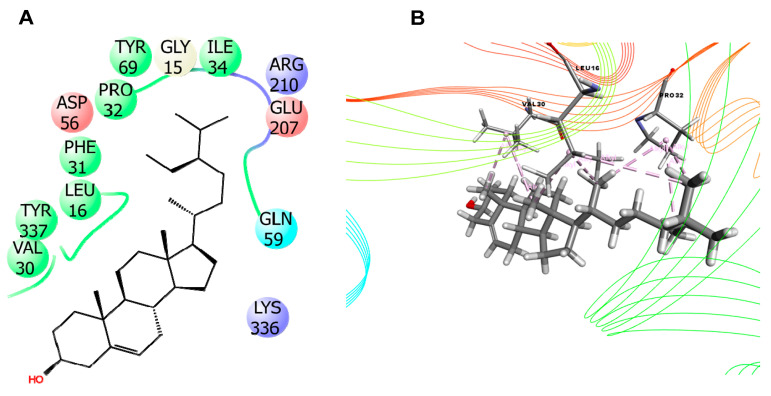
The best 2D (**A**) and 3D (**B**) representation of the interactions between beta-sitosterol with *V. cholerae* MARTX toxin (PDB ID: 3CJB).

In this study, molecular docking was performed as it has been greatly used to predict the ligand-target interactions for gaining better insights into pharmacological activity of natural products [36]. For determining better perceptions of the observed biological activity (anti-inflammatory, analgesic, anti-diarrheal, anxiolytic, locomotor and sedative) of *B. pilosa*, one compound (beta-sitosterol) was selected for the docking analysis where the result of docking study are shown in Table 7. The present study showed that beta-sitosterol displayed −5.01 kcal/mol docking score against the PDE4 (PDB ID: 4WCU) for anti-inflammatory activity. In the case of analgesic activity, beta-sitosterol did not posses any interaction against the COX1 receptor (PDB ID: 2OYE), whereas −3.865 kcal/mol docking score was exhibited against the COX2 (PDB ID: 6COX) enzyme. The compound displayed −2.041 Kcal/mol and −4.273 kcal/mol docking score against the potassium channel (PDB ID: 4UUJ) and bromodomain of human BRD4 in complex with midazolam (PDB ID: 3U5K), respectively, for anxiolytic activity. In addition, it was found to possess the highest docking score of −6.656 kcal/mol against serotonin transporter (PDB ID: 5I6X) for locomotor activity, while exhibiting −4.494 kcal/mol docking score against bromodomain of human BRD4 (PDB ID: 3U5J). Furthermore, for anti-diarrhoeal docking analysis, beta-sitosterol showed −2.01 kcal/mol against the *Vibrio cholerae* MARTX toxin (PDB ID: 3CJB). The results of binding interactions of the docking study are presented in Table 8 and Figure 5, Figure 6 and Figure 7. Here, beta-sitosterol interacts with the 6COX through three hydrogen bonds and twelve hydrophobic interactions as shown in Table 8; 5I6X through two hydrogen bonds and 11 hydrophobic interactions; 4WCU through 12 hydrophobic interactions; 4UUJ through one hydrogen bond and three hydrophobic interactions; 3U5K through nine hydrophobic interactions; 3U5J through 14 hydrophobic interactions and 3CJB through nine hydrophobic interactions. Of all the receptors, 6COX, 5I6X and 4UUJ showed the better binding affinity in both hydrogen bond and hydrophobic bonds interaction.

Moreover, to support our experimental studies, a computer based online tool “PASS” was used to predict different biological activity based on the structure of the compounds. In this study, the possible biological activities of beta-sitosterol were evaluated by the PASS online program (Table 9). The results of this study were defined as Pa (probable activity) and Pi (probable inactivity), where the values of both Pa and Pi should be ranging from 0.000 to 1.000. The Pa value of beta-sitosterol for anti-nociceptive, anti-inflammatory, antisecretory, neurotransmitter uptake inhibitor, neurotrophic factor enhancer and neuropeptide Y4 antagonist activities were 0.558, 0.467, 0.427, 0.266, 0.218 and 0.288, respectively. This outcome is consistent with our experimental studies which exhibited that MEBPL has anti-nociceptive, anti-inflammatory and neuropharmacological activities, including other biological properties.

The bioactive compound (beta-sitosterol) was then further characterized by using ADME analysis to pursue the physiochemical, drug-likeness and pharmacokinetics characteristics. The absorption, distribution, metabolism and excretion (ADME) properties of beta-sitosterol for selective activity was assessed with the QikProp module of Schrodinger (Table 10). The predicted properties of beta-sitosterol satisfied the Lipinski’s rules of five [37] where only one rule (Log P) of violation was observed.

## 3. Materials and Methods

### 3.1. Plant Collection

On March 2019, fresh green leaves of *B. pilosa* were collected from Bhatiari, Chittagong, Bangladesh (GPS coordinates: 22°28′4.7604′′ N and 91°43′38.7300′′ E). The plant was identified by Sheikh Bokhtear Uddin, Department of Botany, University of Chittagong, Chittagong-4331, Bangladesh (accession number: 36186).

### 3.2. Preparation of Plant Extract

The green leaves were dried for 10 days below shade and floor. The ground leaves (310 mg) were soaked in adequate amount of methanol (95%). Importantly, methanol is known as the maximum common solvent that is used to extract most of the phyto-constituents found in natural materials. During the extraction, amber glass was used to keep methanol-soaked powder of the plant leaves and stored at 25 °C/room-temperature and permitted to draw for numerous days (5–7) with continuous shaking and stirring. When the solvent become concentrated, the contents were decanted using cotton and then filtered via Whatman clear out paper No. 1. The solvent was evaporated with the help of a water tub to produce a viscous mass and then kept at room temperature.

### 3.3. Chemicals

All residual chemicals were of analytical grade. Hydrocortisone, bovine albumin serum, diclofenac sodium, diazepam and thiopental-Na were purchased from Square Pharmaceuticals Ltd. (Dhaka, Bangladesh), loperamide was collected from Beacon Pharmaceuticals (Dhaka, Bangladesh). The other chemicals like phosphate buffer, acetic acid and formalin were brought from local sources.

### 3.4. Experimental Animals

Animals (Swiss albino mice) of both sexes (25–35 g) were procured from the Jahangirnagar University, Dhaka-1342, Bangladesh. The animals were housed according to standard guidelines (room temperature, 25 ± 2 °C; relative humidity, 55–60%, 12 h light/dark cycle) and provided sufficient meal and water supplies. For the in vivo tests, the mice were managed with the laboratory conditions for 14 days. The institutional animal ethical committee, Department of Pharmacy, International Islamic University Chittagong, Bangladesh approved the experimental study as stated by the governmental guidelines under the reference Pharm-P&D-61/08′16-130 [38].

### 3.5. Acute Toxicity Study

Mice were separated into four groups of five mice in each. They were fasted overnight and then intraperitoneally administered MEBPL at the doses of 1000, 2000 and 3000 mg/kg, the only vehicle given to the mice of the control group. The animals were observed for three hours for any bizarre behaviour which included respiratory complexity, motor impairment and hyperexcitability. Moreover, the prevalence of mortality for individual groups was noted as much as 24 h after management [39].

### 3.6. Phytochemical Screening

The phytoconstituents analysis of the methanol extract of *B. pilosa* leaves was done by following the previously described standard process. The secondary metabolites such as alkaloids, carbohydrates, glycoside, flavonoids, phenols, tannins, saponins, terpenoids, cholesterols, proteins, fixed oils, quinones and resins were determined [40].

### 3.7. In Vitro Anti-Inflammatory Activity

#### 3.7.1. Membrane Stabilization

Membrane stabilization activity of MEBPL was done by following a previously described method [41]. The test solution contained 0.5 mL of dissimilar concentrations (62.5, 125, 250 and 500 µg/mL) of MEBPL with 1 mL of buffer (phosphate) solution, 2 mL of hyposaline and 0.5 mL of suspension of HRBC (human red blood cell). For the standard (diclofenac sodium), the same procedure was followed. The control solution contained all the chemicals except the extract and reference drug. Then the mixtures were placed in an incubator at 37 °C for 30 min, and followed by centrifugation at 3000 rpm for 20 min. The supernatant solutions were collected and the haemoglobin content in the supernatant solution was measured at 560 nm using UV-visible spectrophotometer. The membrane stabilization of MEBPL was calculated by using the following formula (Equation (1)):

(1) Membrane stability = ((A1 − A2)/A1) × 100

where *A*1 = control absorbance, and *A*2 = sample absorbance.

#### 3.7.2. Inhibition of Protein Denaturation

Anti-inflammatory activity of the methanol extract of *B. pilosa* leaves was carried by the inhibition of albumin-protein denaturation [42]. The test solution (0.5 mL) consisted of 0.45 mL of 0.5% *w*/*v* aqueous solution of bovine serum albumin and sample (0.05 mL) of dissimilar doses (62.5, 125, 250 and 500 µg/mL). Furthermore, 0.5 mL of control solution was also prepared (0.45 mL of 0.5% w/v bovine serum albumin + 0.05 mL of d.w.). Using 1 N HCl, pH was adjusted to 6.3 to all solutions including MEBPL and diclofenac sodium. All the test solutions were then kept at 37 °C in an incubator for 20 min and then the temperature was raised to 57 °C for 3 min. After cooling the sample, 2.5 mL of phosphate buffer was added to the above sample solutions. With the help of the ultraviolet (UV)-spectrophotometer, the absorbance was recorded at 416 nm. Inhibition percentage of albumin-protein denaturation was calculated as (Equation (2)):

(2) Percentage inhibition of protein (albumin) =((A1 – A2)/A1) × 100

where *A*1 = control absorbance, and *A*2 = sample absorbance.

### 3.8. In Vivo Analgesic Activity

#### 3.8.1. Writhing Inhibition Test by Inducing Acetic Acid

Analgesic effect of MEBPL was studied by the writhing inhibition of mice after inducing acetic acid [43]. The experimental mice were separated into 4 different groups (*n* = 5) and were unfed for two hours before starting the test. Tween-80 (1%) solution at 10 mL/kg b.w. as negative control, diclofenac sodium (reference drug) at 10 mg/kg b.w. as positive control and doses of MEBPL at 200 and 400 mg/kg b.w., respectively, administered orally using gavage. After 30 min of administration, acetic acid (0.7%) was injected intraperitoneally into the treated animals and then the writhing response was noted for 20 min.

#### 3.8.2. Paw Licking Test by Inducing Formalin

Paw licking test by inducing formalin was carried out for the study of analgesic activity by using the previously described method [44]. The management and treatment of each group (*n* = 5) of mice were described in the writhing inhibition test. After 30 min of the administration of sample dose, at the right hind paw of the mice, 20 μL formalin (2.5% *v*/*v*) was injected by using a micro syringe (26-guage needle) at the bottom of the dorsal surface of the skin. The early phase (first 5 min) paw licking and the late phase (15–30 min) paw licking was recorded for the calculation.

### 3.9. Anxiolytic Activity by Hole Board Experiment

Hole-board study was done to determine the anxiolytic activity of the plant extract [45]. The apparatus consisted of 16 evenly distributed holes in a plane wooden space (40 × 40 cm^2^) in diameter. The mice were separated into four groups (*n* = 5). Group 1 treated with negative control (1% Tween-80), group 2 treated with positive control (diazepam 1 mg/kg), groups 3 and 4 received the plant extract dosages of 200 and 400 mg/kg, respectively. Thirty minutes after oral administration of vehicle or MEBPL and 15 min after administration of diazepam, respectively, every animal was positioned at the middle of the wooden space to freely move and the amount of dipping head into the holes was observed for each mouse and recorded for 5 min.

### 3.10. Locomotor Activity by Open Field Experiment

In the open field test of locomotor activity, the mice were separated into four groups containing five mice in each. The dosing of each group was carried out as described in anxiolytic section. After dosing, each mouse was placed into a four-sided box of 25 equivalent black and white coloured squares with dimensions of 60 × 60 × 60 cm^3^, for 3 min at 0, 30, 60, 90 and 120 min intervals and the movements were recorded [46].

### 3.11. Sedative Activity by Thiopental-Sodium Induced Sleeping Time

The experimental mice were again separated into four groups (*n* = 5). The dosing was carried out as in the anxiolytic section. After 30 min of dosing, each mouse was treated with thiopental-Na (40 mg/kg b.w.) orally to induce sleep. The time from administration of thiopental-Na to loss of righting reflex (latent period) and also the time of the loss and retrieval of righting reflex (duration of sleep) were observed. The onset time of sleeping and the total duration of sleeping were noted for each mouse [47].

### 3.12. Anti-Diarrheal Activity

#### 3.12.1. Castor-Oil Induced Diarrhoea Test

To evaluate the anti-diarrheal effect of MEBPL, the method of castor-oil induced diarrhoea test was followed as described previously [48]. The mice were separated into four groups (*n* = 5) and remained unfed for 24 h. Mice from groups 1, 2, 3 and 4, respectively, received 10 mL/kg of 1% Tween-80 orally as negative control, standard drug loperamide (5 mg/kg) as positive control, doses of 200 mg/kg b.w. of MEBPL and doses of 400 mg/kg b.w. of MEBPL. After 1 h, 1 mL castor oil was given orally to every mouse and they were placed separately into boxes of blotting white paper. For each mouse, the consistency and number of both wet and dry faecal drops were observed as well as noted after each hour interval over a 4-h period. The paper was changed after every hour. Afterward, the faeces (means) of the treated groups were compared to the control group and the percentage of inhibition of defecation was calculated using the following equation (Equation (3)):

(3) % of inhibition of defecation = [(A − B)/A] ×100

where *A* = average of faeces number (control group), and *B* = average of faeces number (treated group).

#### 3.12.2. Gastro-Intestinal Motility Test

The previously described method by Mascolo et al. [49] was used to carry gastro-intestinal motility experiment. The treatment was followed as described in the castor oil-induced diarrhoea test. Charcoal feed was prepared by mixing 10% charcoal with 5% gum-acacia and after 1 h of administration of the sample, 1 mL charcoal feed was given orally to each animal. After 1 h of application of charcoal, the mice were forgone and the length (cm) moved by the charcoal feed from the pylorus to caecum was recorded. The subsequent equations were used to calculate the percentage of inhibition and peristalsis index (Equations (4) and (5)):

(4) % of inhibition = [(X − Y)/X] ×100

(5) Peristalsis index  = (Y/X) ×100

where *X* = distance travelled by the charcoal for control (cm), and *Y* = distance travelled by the charcoal for treated group (cm).

### 3.13. In Silico Studies

#### 3.13.1. Selection of Compounds for In Silico Studies

Phytosterol is a steroidal compound having a similar structure to cholesterol, and more than 200 different types of sterols have been separated and isolated from different plants. Phytosterols are basically used as food supplements that having a cholesterol-lowering property. Phytosterols are available in the market in the form of capsules and tablets with other multivitamins. The average intake of naturally occurring phytosterols ranges between 150 and 450 mg/day. Beta-sitosterol is one of the naturally occurring phytosterols having steroidal moiety. Beta-sitosterol reduces cholesterol levels by competing with cholesterol for absorption in the intestine, and due to these aspects, it is valuable for cardiovascular protection. Beta-sitosterol also inhibits tumour growth, modulates the immune response, and has antioxidant capacity. In the future, beta-sitosterol could be a potential chemo-preventive agent for the treatment of various types of cancer, including prostatic carcinoma and breast cancer [20].

In the present study, we have collected data regarding detailed pharmacological activities and analytical aspects of beta-sitosterol, which could be beneficial to the researchers, scientist including in the medical and pharmaceutical fields. Moreover, there are no previous reports on isolated compounds from *B. pilosa* to date except the isolation of beta-sitosterol. From this perspective, we have selected beta-sitosterol for computational studies such as molecular docking, PASS prediction, and ADME analysis.

#### 3.13.2. Molecular Docking Analysis

Beta-sitosterol was isolated from the roots of *B. pilosa* which was subjected molecular docking. The docking analysis was followed by the protocol of Sastry et al. (2013) [50] and explained in detail in our previous studies [11,51]. Protein data bank was used to derive the 3D structures of the subsequent proteins: cyclooxygenase (COX)-1 (PDB ID: 2OYE) [52], cyclooxygenase (COX)-2 (PDB ID: 6COX) [53], serotonin transporter (PDB ID: 5I6X) [54], potassium channel (PDB ID: 4UUJ) [55], PDE4 complexed with inhibitor (PDB ID: 4WCU) [56], bromodomain of human BRD4 (PDB ID: 3U5J) [57], *V. cholerae* MARTX toxin and (PDB ID: 3CJB) [58], and bromodomain of human BRD4 in complex with midazolam (PDB ID: 3U5K) [57]. The molecular docking analysis was assessed by Schrodinger Maestro version 11.1 and the amino acid residues of the active sites of each receptor were given as follows: for 2OYE receptor, the amino acid residues were Val116, Arg120, Glu524, Leu531, Ala527, Gly526, Trp387, Met522, Leu384, Tyr385, Ser530, Val349, Leu352, Ile523, Ser353, Phe518, Tyr355, and Leu93; for 6COX receptor, the amino acid residues were Trp387, Tyr385, Gly526, Ala527, Leu384, Met522, Leu352, Phe518, Ala516, Gln192, Arg513, Ser353, Tyr355, Val116, Arg120, Leu531, Leu359, Val349, Val523, and Ser530; for 5I6X receptor, the amino acid residues were Val501, Phe335, Gly338, Ser336, Asp98, Ala96, Tyr95, Tyr176, Ser438, Ser439, Ile172, Ala169, Gly442, and Phe341; for 4UUJ receptor, the amino acid residues were Val93, Arg89, and Leu86; for 4WCU receptor, the amino acid residues were Phe432, Met273, Thr271, Asp318, Leu319, Tyr159, Trp332, Asn321, Thr333, Ile336, Phe340, Met337, Gln433, Gln369, Met357, Ile376, Phe372, and Leu436; for 3U5J receptor, the amino acid residues were Pro82, Trp81, Leu92, Met149, Ile146, Leu94, Asn140, and Phe83; for 3CJB receptor, the amino acid residues were Gly158, Leu16, Val159, Gly13, Gly15, Ser14, Gly302, Lys336, Tyr306, Met305, Glu214, Lys213, Thr303, Asp157, Gly301, Lys18, Gly156, and Asp154; and for 3U5K receptor, the amino acid residues were Asn140, Leu94, Leu92, Met149, Ile146, Trp81, Pro82, Val87, and Phe83.

#### 3.13.3. PASS Prediction Study

The beta-sitosterol (PubChem ID: 222284) was also used for PASS prediction analysis and the chemical structure for the compound was retrieved from the PubChem data base. PASS online server predicts the pharmacological activity of a compound as probable activity (Pa) and probable inactivity (Pi) and details were described in our previous study [11].

#### 3.13.4. ADME Analysis

The pharmacokinetics parameters of the beta-sitosterol compound was analysed based on Lipinski’s rule of five and assessed by QikProp (Schrodinger Maestro, version 11.1) [59]. This application can measure the pharmacokinetic and physicochemical properties with molecular weight, hydrogen bond donor, hydrogen bond acceptor, high lipophilicity (Log P) and rotatable bonds of compounds.

### 3.14. Statistical Analysis

The values were presented in mean ± SEM (standard error mean), where ^a^ *p* < 0.05, ^b^ *p* < 0.01, ^c^ *p* < 0.001 and ^d^ *p* < 0.0001 were considered as statistically significant. The statistical analysis followed by one-way analysis of variance ANOVA (Dunnett’s test) compared to the negative control (1% Tween-80) using GraphPad Prism version 6.0 (GraphPad Software Inc., San Diego, CA, USA).

## 4. Conclusions

Briefly, the experimental evidences support the medicinal value and potentiality of *B. pilosa.* Our study provides notable proof of significant and dose dependent anti-inflammatory activity of MEBPL. The experimental study also showed that MEBPL exhibited noticeable analgesic activity in different pain models. It has also been revealed that MEBPL possesses remarkable anxiolytic, locomotion and sedative effect. Furthermore, expressive anti-diarrheal activity increases the importance of this plant. In addition, molecular docking, PASS prediction and ADME analysis revealed that the selective compound beta-sitosterol could be a potential candidate for the named biological activity. Therefore, *B. pilosa* can be considered as a viable candidate for the development of new drugs as it has numerous pharmacological activities. However, to explore its biological activity and mechanisms on animals and humans necessitates further extensive studies.

## Figures and Tables

**Table 1:** Qualitative phytochemical screening of methanol extract of *B. pilosa* leaves.

Phytochemical Test	Results
Alkaloids	++
Carbohydrates	+
Glycosides	++
Flavonoids	++
Phenols	+
Tannins	+
Saponins	+
Terpenoids	+
Cholesterol	-
Protein	-
Fixed oil	+
Quinones	+
Resins	+

Here, (++) = highly present, (+) = moderately present, and (-) = absent.

**Table 2:** Analgesic activity of methanol extract of *B. pilosa* leaves (MEBPL) in acetic acid induced writhing inhibition test in Swiss albino mice.

Treatment	Writhing Number	(%) Inhibition
Control	37.20 ± 2.46	-
Diclofenac-Na (10 mg/kg)	12.80 ± 0.97 ^c^	65.59
MEBPL (200 mg/kg)	28.00 ± 1.79 ^a^	24.73
MEBPL (400 mg/kg)	24.60 ± 2.44 ^b^	33.87

Values are given as mean ± SEM (*n* = 5). ^a^ *p* < 0.05, ^b^ *p* < 0.01 and ^c^ *p* < 0.001 compared with control group followed by Dunnett’s test of one-way analysis of variance ANOVA (GraphPad Prism 6.0).

**Table 3:** Effect of analgesic activity of methanol extract of *B. pilosa* leaves (MEBPL) by formalin induced paw induced test on Swiss albino mice.

Treatment	Early Phase Paw Licking (s)	% Inhibition of Early Phase	Late Phase Paw Licking (s)	% Inhibition of Late Phase
Control	57.80 ± 3.02	-	46.20 ± 1.74	-
Diclofenac-Na (10 mg/kg)	17.40 ± 1.12 ^d^	69.89	16.00 ± 1.14 ^d^	65.37
MEBPL (200 mg/kg)	40.40 ± 2.25 ^b^	30.11	35.20 ± 2.69 ^b^	23.81
MEBPL (400 mg/kg)	36.00 ± 3.85 ^c^	37.72	29.40 ± 2.84 ^c^	36.36

Values are given as mean ± SEM (*n* = 5). ^b^ *p* < 0.01, ^c^ *p* < 0.001 and ^d^ *p* < 0.0001 compared with control group followed by Dunnett’s test of one-way ANOVA (GraphPad Prism 6.0).

**Table 4:** Anxiolytic effect of methanol extract of *B. pilosa* leaves (MEBPL) via hole-board test in mice.

Treatment	Number of Head Dipping
Control	24.80 ± 3.07
Diazepam 1 mg/kg	58.20 ± 4.02 ^d^
MEBPL 200 mg/kg	30.60 ± 2.42
MEBPL 400 mg/kg	40.20 ± 2.82 ^b^

Values are stated as mean ± SEM (*n* = 5). ^b^ *p* < 0.01 and ^d^ *p* < 0.0001 compared with control group followed by Dunnett’s test of one-way ANOVA (GraphPad Prism 6.0). MEBPL = methanol extract of *B. pilosa* leaves.

**Table 5:** The anti-diarrheal effect of methanol extract of *B. pilosa* leaves (MEBPL) on castor-oil induced Swiss albino mice.

Treatment	Total Number of Faeces	% Inhibition of Defecation	Total Number of Diarrheal Faeces	% Inhibition of Diarrhoea
Control	14.70 ± 0.41	-	7.05 ± 0.25	-
Loperamide (5 mg/kg)	5.55 ± 0.46 ^d^	62.24	2.05 ± 0.15 ^c^	70.92
MEBPL (200 mg/kg)	6.55 ± 0.56 ^d^	55.44	2.30 ± 0.22 ^c^	67.38
MEBPL (400 mg/kg)	5.50 ± 0.34 ^d^	62.59	1.80 ± 0.33 ^d^	74.46

Values are given as mean ± SEM (*n* = 5). ^c^ *p* < 0.001 and ^d^ *p* < 0.0001 compared with control group followed by Dunnett’s test of one-way ANOVA (GraphPad Prism 6.0). MEBPL = methanol extract of *B. pilosa* leaves.

**Table 6:** Intestinal motility effect of methanol extract of *B. pilosa* leaves by using charcoal as a marker.

Treatment	Total Length of Intestine (cm)	Distance Travel by Charcoal (cm)	% Peristalsis Index	% Inhibition
Control	52.80 ± 1.59	44.20 ± 1.66	83.92 ± 3.55	-
Loperamide (5 mg/kg)	53.20 ± 1.93	24.20 ± 1.88 ^d^	45.33 ± 2.27 ^d^	54.51
MEBPL (200 mg/kg)	55.60 ± 3.31	31.80 ± 1.66 ^c^	57.93 ± 4.44 ^c^	42.80
MEBPL (400 mg/kg)	57.80 ± 2.15	29.40 ± 2.29 ^c^	51.30 ± 5.04 ^c^	49.13

Values are given as mean ± SEM (*n* = 5). ^c^ *p* < 0.001 and ^d^ *p* < 0.0001 compared with control group followed by Dunnett’s test of one-way ANOVA (GraphPad Prism 6.0). MEBPL = methanol extract of *B. pilosa* leaves.

**Table 7:** Molecular docking score of beta-sitosterol isolated from *B. pilosa* leaves.

Compound	4WCU	2OYE	6COX	4UUJ	3U5K	5I6X	3U5J	3CJB
Beta-sitosterol	−5.01	-	−3.865	−2.041	−4.273	−6.656	−4.494	−2.01

Here, 4WCU PDB ID for anti-inflammatory activity; 2OYE and 6COX PDB ID for analgesic activity; 4UUJ and 3U5K PDB ID for anxiolytic activity; 5I6X PDB ID for locomotor activity; 3U5J PDB ID for sedative activity; and 3CJB PDB ID for anti-diarrheal activity.

**Table 8:** Binding interactions of the beta-sitosterol with the respective proteins for different biological activities.

Receptor PDB ID	Hydrogen Bond Interactions	Hydrophobic Bond Interactions
6COX	ASN 382 (2), THR 212	HIS 207 (Pi-Alkyl), HIS 214 (Pi-Alkyl), HIS 386 (Pi-Alkyl), HIS 388 (Pi-Alkyl), PHE 404 (Pi-Alkyl), VAL 291(2) (Alkyl), VAL 444 (Alkyl), VAL 447 (Alkyl), LEU 391 (Alkyl), LEU 408 (Alkyl), ALA 443 (Alkyl)
5I6X	ARG 104	PHE 355(2) (Pi-Alkyl), PHE 341(2) (Pi-Alkyl), TYR 176(2) (Pi-Alkyl), ILE 172(3) (Alkyl), ALA 169 (Alkyl), ALA 173 (Alkyl)
4WCU	-	ILE 336 (Alkyl), LEU 319 (Alkyl), MET 273(2) (Alkyl), PHE 340 (Pi-Alkyl), PHE 372 (Pi-Alkyl), PHE 432(2) (Pi-Alkyl), TYR 159(2) (Pi-Alkyl), HIS 164 (Pi-Alkyl), HIS 160 (Pi-Alkyl)
4UUJ	THR 72	TYR 82 (3) (Pi-Alkyl)
3U5K	-	TYR 139(2) (Pi-Alkyl), TYR 97 (Pi-Alkyl), PHE 83(2) (Pi-Alkyl), VAL 87 (Alkyl), LEU 94 (Alkyl), PRO 82 (Alkyl), CYS 136 (Alkyl)
3U5J	-	VAL 87(2) (Alkyl), LEU 92 (Alkyl), LEU 94(2) (Alkyl), PRO 82(2) (Alkyl), ILE 146(3) (Alkyl), TRP 81 (Pi-Alkyl), TYR 139 (Pi-Alkyl), TYR 97(2) (Pi-Alkyl)
3CJB	-	VAL 30(3) (Alkyl), PRO 32(3) (Alkyl), LEU 16(3) (Alkyl)

**Table 9:** PASS prediction of the beta-sitosterol isolated from *B. pilosa* leaves.

Properties	Pa	Pi
Antinociceptive	0.558	0.014
Anti-inflammatory	0.467	0.067
Antisecretory	0.427	0.049
Neurotransmitter uptake inhibitor	0.266	0.259
Neurotrophic factor enhancer	0.218	0.051
Neuropeptide Y4 antagonist	0.288	0.200

Here, (Pa) probable activity, and (Pi) probable inactivity.

**Table 10:** ADME analysis of beta-sitosterol isolated from *B. pilosa* leaves.

ADME Analysis of Beta-Sitosterol	
Molecular weight (acceptable range: 500)	414.7
Hydrogen bond donor (acceptable range: ≤5)	1
Hydrogen bond acceptor (acceptable range: ≤10)	1
High lipophilicity (expressed as Log P, ˂5)	9.3
Rotatable bond	6

## Abbreviations

MEBPL: Methanol extract of *Byttneria pilosa* leaves; HRBC: Human red blood cell; UV: Ultra-violet; b.w.: Body weight; d.w.: Distilled water; NSAIDs: Non-steroidal anti-inflammatory drugs; PASS: Prediction of Activity Spectra for Substances; ADME/T: Absorption, Distribution, Metabolism, Excretion, and Toxicity; SEM: Standard error mean.
